# Elucidating the Mechanism(s) Underlying Antipsychotic and Antidepressant-Mediated Fractures

**DOI:** 10.29245/2578-2959/2018/1.1106

**Published:** 2017-12-26

**Authors:** Karen L. Houseknecht, C.C. Bouchard, C.A Black

**Affiliations:** 1 College of Osteopathic Medicine, University of New England, 11 Hills Beach Road, Biddeford, ME 04005, USA; 2 College of Pharmacy, University of New England, 11 Hills Beach Road, Biddeford, ME 04005 USA

**Keywords:** Antipsychotic, Selective Serotonin Reuptake Inhibitor, Mood disorder, Fracture, Bone Mineral Density, Risperidone

## Abstract

Mood spectrum disorders and medications used to treat these disorders, such as atypical antipsychotic drugs (AA), are associated with metabolic and endocrine side effects including obesity, dyslipidemia, hyperglycemia and increased risk of fractures. Antidepressant medications, including selective serotonin reuptake inhibitors (SSRI), have also been reported to increase fracture risk in some patients. The pharmacology underlying the increased risk of fractures is currently unknown. Possible mechanisms include alternations in dopaminergic and/or serotonergic signaling pathways. As these medications distribute to the bone marrow as well as to the brain, it is possible that drug-induced fractures are due to both centrally mediated effects as well as direct effects on bone turnover. Given the growing patient population that is prescribed these medications for both on- and off-label indications, understanding the level of risk and the mechanisms underlying drug-induced fractures is important for informing both prescribing and patient monitoring practices.

## Introduction

Mood spectrum disorders are often comorbid with metabolic and endocrine disease. Mental illness, genetic background and drug therapies all contribute to metabolic disease susceptibility, however the relative contribution of these factors is not clear. Antipsychotic medications, originally designed and FDA-approved for the treatment of psychosis associated with schizophrenia and bipolar disorder, are increasingly prescribed off-label for diverse indications, often in vulnerable populations including children and the elderly. Because of the increasing rate of off-label prescribing, these are some of the most highly prescribed drugs, world-wide, resulting in a very large patient population exposed to these powerful psychiatric medications^1–2^. Second generation or atypical antipsychotics (AA) are known to cause endocrine and metabolic side effects including rapid onset obesity, dyslipidemia and diabetes. Clinical evidence is emerging that these medications also increase the risk of fractures in adults and children, resulting in new FDA warning language relating to increased fracture risk on the labels of all antipsychotic medications^3^.

Antidepressant medications, specifically selective serotonin reuptake inhibitors (SSRI), have also been implicated in bone loss in some patients. The skeleton is continuously undergoing remodeling, an energy-requiring process whereby bone is broken down by osteoclast cells followed by deposition of new bone, largely involving osteoblast cells^4^. When the rate of bone remodeling is altered, changes in bone density occur. When bone loss exceeds the rate of bone deposition, bones weaken, ultimately leading to osteoporosis and increased risk of fracture. In this mini-review, we highlight what is known about the impact of mental health and psychiatric medications (AA and SSRI) on bone biology and highlight emerging mechanisms implicated in these adverse side effects.

### Genetic Susceptibility

Individuals with mental illness, particularly, schizophrenia and affective disorders, have a considerably shorter lifespan than the general population^5–8^. Reduced bone mass, especially osteoporosis, is significantly more common in people with schizophrenia, bipolar disorder, and depression than controls^9–11^. Genetic framework and the use of psychotropic medications play a major role in the preeminent risk of physical morbidity and mortality related to these disorders, including metabolic disorders, endocrine diseases, and fractures. Current literature suggests the mechanism of decreased bone mineral density (BMD) in patients with schizophrenia and affective disorders is multifactorial, including genetic predispositions to lower BMD. Low bone mass and microarchitectural deterioration of bone tissue have been shown to be 50–80% genetically determined^12^. In a large prospective study of white American women 65 years of age or older, a history of hip fracture in their mother doubled the risk of hip fracture^13^. A large genomic-wide meta-analysis concluded that 56 genomic loci are associated with BMD variation and 14 loci are associated with increased bone fracture^14^.

### Psychiatric medications and bone: Clinical evidence

Antipsychotic medications have complex pharmacology and are associated with increased falls and fractures across diverse patient populations. As antipsychotic medications cause somnolence and orthostatic hypotension (due to α-adrenergic receptor antagonism), the elevated fracture risk in patients taking antipsychotic drugs has been attributed to an increase in falls, especially in elderly patients. It is not clear, however, if the drug-associated increase in falls is the sole cause of increased fractures, or if other mechanisms are also at play. Specifically, a number of recent cross-sectional and longitudinal clinical studies have found that AA use is associated with reduced BMD and increased fracture risk in both men and women^15–18^. Serum concentrations of a bone turnover marker, β-CrossLaps, were higher in patients treated with AA (n=31 drug-naïve first episode patients and n=85 AA monotherapy for 6 months) vs. drug-naive first-episode patients and normal subjects^19^. A prospective study found that BMD in 120 firstepisode inpatients with schizophrenia prescribed either clozapine, quetiapine or aripiprazole was significantly lower than in matched healthy controls (n=90), 12-months after drug initiation^17^. In pediatric patients, AA-associated fractures were increased 2–3 fold with an associated reduction in bone mass^20–21^. Given the devastating effects of fractures in elderly patient populations, as well as the potential of inhibiting peak bone accrual in pediatric patients, the antipsychotic-induced association with increased fracture risk should impact prescribing practices in vulnerable patient populations.

The effect of antidepressants on BMD is controversial. A population-based cohort study assessed the incidence of non-traumatic major osteoporotic fractures (MOFs) and hip fractures in men and women age 40 or older who had a received a dual-energy x-ray absorptiometry scan^22^. A total of 68,730 individuals were studied, 4,122 of whom were on SSRI therapy. After a median of 6.7 years of observation, it was found that the use of SSRI was independently associated with significantly increased risk for both MOFs and hip fracture. A significant association with SSRI use and lower femoral neck BMD was found in post-menopausal women (118 SSRI vs 1,669 non-SSRI users) 5 years after follow-up^23^. This association was strong and dose-dependent for those who had purchased a SSRI for more than 1 year of use. Recently, a prospective cohort study of 4,915 men and 5,831 post-menopausal women (age > 45 years) with a total of four BMD measurements every 4–5-years post-SSRI exposure, reported no association with loss of BMD after taking the duration of treatment into account^24^. A longitudinal comparison of SSRI and AA use in boys found that chronic SSRI treatment was associated with reduced but stable bone mass, while chronic risperidone (AA) treatment resulted in a failure to accrue bone mass^21^. Given the variability in clinical findings with SSRI therapy, additional longitudinal prospective studies are required to delineate whether SSRI use is associated with BMD.

### Elucidating underlying mechanisms: Hyperprolactinemia

A popular hypothesis in the clinical literature is that the AA-induced increase in fractures is due to hyperprolactinemia induced hypogonadism^20^. It is well known that hypogonadism causes bone loss and increased fracture risk, and antipsychotic medications can cause hyperprolactinemia in women, men and children. Likewise, data in rodent models indicate that AA can cause hyperprolactinemia^25–26^. In order to evaluate mechanism(s) underlying AA-induced bone loss, we developed a pre-clinical model of antipsychotic induced bone loss using the AA drug, risperidone (RIS) ^27^. In this model, young female C57BL6/J mice were treated for 5 or 8 weeks with clinically relevant doses of RIS (0.75 mg/kg BW), commencing at 3.5 weeks of age. RIS treatment resulted in reduced trabecular bone density and volume^27^. Likewise, it has been reported that RIS-treated mice had significantly lower trabecular bone density and volume, bone volume/total volume, connectivity, and trabecular number relative to controls^28^. In order to test the hypothesis that RIS induced bone loss is due to hypogonadism, ovariectomized (OVX) and sham-operated mice were used to determine if RIS treatment (8 weeks) could impact bone density^26^. It was found that OVX and RIS alone caused bone loss. Furthermore, RIS and OVX in combination resulted in even greater bone loss than observed with either treatment alone, indicating that hyperprolactinemia-induced hypogonadism is not sufficient to explain the AA-induced bone loss.

### Elucidating underlying mechanisms: Central effects

It has not been determined if the detrimental effects of psychiatric medications on bone biology are mediated centrally, or are due, at least in part, to direct effects on bone. AA and SSRI medications distribute to the CNS and elicit their effects on mood via central mechanisms. AA drugs have complex pharmacology, posing as potent antagonists or inverse agonists at diverse G-protein coupled receptor families including dopamine, serotonin, a-adrenergic, histamine and muscarinic receptors^29^. Clinical efficacy for AA drugs is thought to be primarily due to dopamine receptor antagonism coupled with serotonin receptor antagonism. It has been hypothesized by several groups that serotonin signaling, and to a lesser extent, dopamine signaling, are implicated in the regulation of bone biology, both at the level of the CNS and locally^30–33^. It is known that bone turnover is regulated by the sympathetic nervous system and it has been hypothesized that serotonin plays a central role in regulating the effects of the hormone, leptin, on bone biology, via regulation of sympathetic output to bone^31–34^.

We hypothesized that regulation of sympathetic output to bone could also be involved in AA-induced bone loss. Pre-clinical studies show that RIS induced bone loss in mice can be rescued with co-administration of the β-blocker, propranolol^35^. Interestingly, the SSRI medication, fluoxetine, has also been reported to cause bone loss, both clinically and in rodent models, and fluoxetine effects can also be rescued by co-administration with propranolol^36^. Although the molecular pharmacological targets of AA and SSRI drugs differ, the fact that both impact serotonergic signaling, and that drug-induced effects on bone can be ameliorated with co-administration of propranolol, gives strong support for central serotonergic signaling pathways playing a role in the regulation of sympathetic tone and down-stream regulation of bone biology. It will be important to determine if the effect of β-adrenergic receptor antagonism of AA and SSRI-induced bone loss is translatable to the clinical setting.

### Elucidating underlying mechanisms: Direct effects

Bone is extensively innervated with both sensory and parasympathetic neurons^34–37^ and thus bone and the bone marrow compartment are exposed to dopamine norepinephrine, epinephrine, and their metabolites^38–39^. Furthermore several laboratories have reported evidence of dopaminergic and serotonergic receptor gene expression in primary osteoblast and osteoclast cells as well as MC3T3 cells which differentiate into osteoblasts^26,40–42^. Dopamine is present in marrow and inhibits osteoclastogenesis and osteoblastogenesis in both primary human osteoclasts and mouse primary osteoclasts and osteoblasts^26,41,43^. Furthermore, the effect of dopamine to inhibit osteoclastogenesis in murine cells was antagonized by the dopamine antagonist, risperidone, further confirming a direct effect of dopamine and AA medications on bone biology^26^. Though these *in vitro* results are compelling, it is important to recognize the numerous *in vivo* factors which influence bone health that are not present in *in vitro* models.

In support of this hypothesis, we quantified clinically relevant concentrations of the AA medication, RIS, and its active metabolite (9-OH RIS) in marrow of mice collected 1–3 hours following a single, clinically relevant oral dose^26^. In fact, the ratio of RIS in marrow vs. blood exceeded 10, indicating drug accumulation in marrow. We have also quantified concentrations of aripiprazole and olanzapine (AA drugs) and fluoxetine (SSRI) in mouse marrow following oral dosing, albeit at lower concentrations than RIS^48^. A clinical report using Magnetic Resonance Spectrometry (MRS) indicated that SSRI medications fluoxetine and fluvoxamine accumulated in human marrow and were present in marrow for months following treatment, long after drug disappearance from the brain and blood compartments, supporting the notion that drug exposure in marrow may have clinical relevance for bone turnover^44^. Osteoblasts and osteoclasts can synthesize serotonin, express the serotonin transporter (SERT), and recent studies in rodents indicate that serotonin plays an inhibitory role in osteoblast function^45–47^. Taken together, these data indicate that at least a portion of the adverse effects of AA and SSRI medications (and their metabolites) may be due to direct drug effects, via dopamine and/or serotonergic receptors expressed on bone cells.

## Conclusions

**Figure 1.**
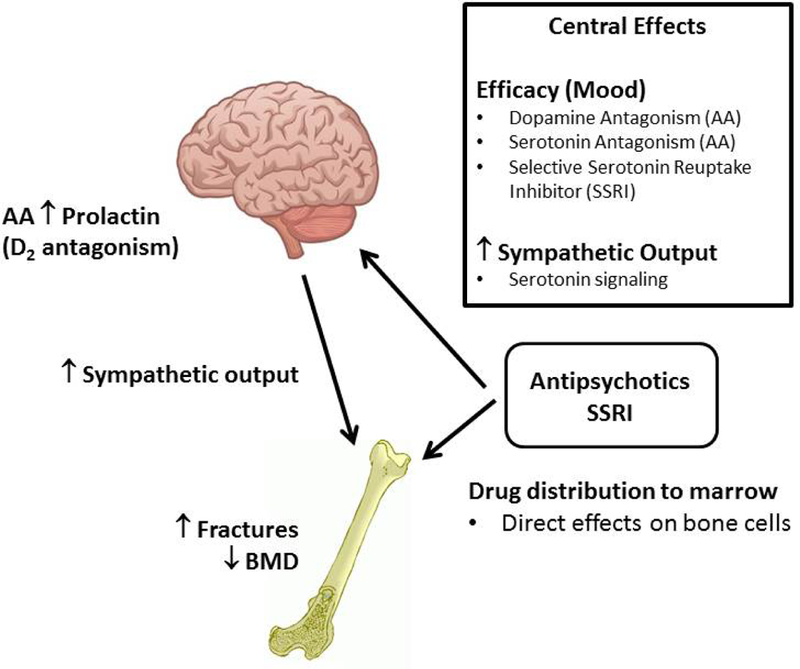
Antipsychotic (AA) and selective serotonin reuptake inhibitor (SSRI) effects on bone are mediated by central and direct mechanisms. AA and SSRI drugs distribute to the brain and to the bone marrow where they elicit effects on behavior, sympathetic tone and direct effects on osteoclast and osteoblast differentiation, all of which contribute to bone loss and increased fracture rate.

Mood spectrum disorders and medications used to treat those disorders are associated with increased metabolic burden for patients and an increase in fracture risk. The mechanistic pharmacology underlying antipsychotic and SSRI-induced fracture risk and bone loss is complex, involves both centrally mediated and direct effects on bone, may include drug accumulation in the marrow compartment, and likely involves dopaminergic, serotonergic and sympathetic signaling systems (Figure 1). Shared mediators intervene in the regulation of the function of both bone and nervous system cells. The existence of these integrated systems partially explains, at the biological level, the interference between neuropsychiatric disorders and bone remodelling, as well as the osteoresorptive effect of some antipsychotic and antidepressant drugs^49,50^. Given the large patient population which is prescribed these medications and the enhanced risk for vulnerable populations such as children and the elderly, prescribing practices for off-label use as well as patient monitoring for metabolic and fracture risk should be carefully evaluated.
